# Implementing a Dementia‐Friendly Communities programme across local authorities in the East of England

**DOI:** 10.1002/alz.088803

**Published:** 2025-01-09

**Authors:** Greg Windle

**Affiliations:** ^1^ University of Hertfordshire, Hatfield, Hertfordshire United Kingdom

## Abstract

There are many types of Dementia‐Friendly Communities (DFCs), and communities define and implement them in different ways. Toolkits from the World Health Organisation and Dementia Friendly America have defined specific goals for DFCs, and in 2013, Alzheimer’s Society created a national recognition programme for UK DFCs to respond to the diversity of interpretations. Key elements of the programme included People (awareness and training), Process (support and signposting) and Place (physical support and community). The complex implementation of DFCs in diverse regions has not been extensively studied, and the Alzheimer’s Society DFC accreditation programme for England ended in 2023.

In order to keep continuity, regional governments in England are developing their own frameworks for implementing DFCs. County‐level local governments have convened a number of district councils to agree to continue with a DFC scheme in 2024 as a key part of the delivery of the county‐level dementia strategy for 2023 through 2028. This dementia strategy covers a broad range of community concerns, but it does not consider detailed implementation questions. A task force made up of local government officials, clinical commissioning group representatives, admiral nursing representatives, care home leaders and voluntary organisation heads is developing and implementing the new DFC programme. The continued DFC programme takes a federal structure, with a county‐wide set of criteria for defining levels of dementia‐friendly services in the community. Each local government within the county then has flexibility to tailor the criteria to their own population, business and service landscape.

As DFCs in the East of England adjust to this new administrative framework, we are using an evaluation and implementation framework to measure individual and community impact and describe implementation consideration for this method of DFC administration. The main focus of implementation science has been in clinical practice, but there is a growing focus on the importance of evidence‐based public policies and their implementation. In detailing the implementation of this innovative community care programme, we consider implementation domains of Contextual Factors, Implementation Strategies, Service and Patient Outcomes, Implementation Outcomes, Economic Evaluation, Stakeholder Involvement and Engagement and Patient and Public Involvement and Engagement.

